# Dating Pupae of the Blow Fly *Calliphora vicina* Robineau–Desvoidy 1830 (Diptera: Calliphoridae) for Post Mortem Interval—Estimation: Validation of Molecular Age Markers

**DOI:** 10.3390/genes9030153

**Published:** 2018-03-09

**Authors:** Barbara K. Zajac, Jens Amendt, Marcel A. Verhoff, Richard Zehner

**Affiliations:** Institute of Legal Medicine, Goethe University, 60596 Frankfurt, Germany; amendt@em.uni-frankfurt.de (J.A.); verhoff@med.uni-frankfurt.de (M.A.V.); zehner@em.uni-frankfurt.de (R.Z.)

**Keywords:** forensic entomology, digital age determination, qPCR, transcriptome analysis, metamorphosis, forensics, blow fly

## Abstract

Determining the age of juvenile blow flies is one of the key tasks of forensic entomology when providing evidence for the minimum post mortem interval. While the age determination of blow fly larvae is well established using morphological parameters, the current study focuses on molecular methods for estimating the age of blow flies during the metamorphosis in the pupal stage, which lasts about half the total juvenile development. It has already been demonstrated in several studies that the intraspecific variance in expression of so far used genes in blow flies is often too high to assign a certain expression level to a distinct age, leading to an inaccurate prediction. To overcome this problem, we previously identified new markers, which show a very sharp age dependent expression course during pupal development of the forensically-important blow fly *Calliphora vicina* Robineau–Desvoidy 1830 (Diptera: Calliphoridae) by analyzing massive parallel sequencing (MPS) generated transcriptome data. We initially designed and validated two quantitative polymerase chain reaction (qPCR) assays for each of 15 defined pupal ages representing a daily progress during the total pupal development if grown at 17 °C. We also investigated whether the performance of these assays is affected by the ambient temperature, when rearing pupae of *C. vicina* at three different constant temperatures—namely 17 °C, 20 °C and 25 °C. A temperature dependency of the performance could not be observed, except for one marker. Hence, for each of the defined development landmarks, we can present gene expression profiles of one to two markers defining the mentioned progress in development.

## 1. Introduction

Development, behavior, and diet of insects can be evidence of forensic issues. Using the knowledge about insects to provide clues about the minimum post mortem interval (min PMI), the minimum time passed since death [1,2,3,4], is the main task of forensic entomology. Blow flies (Calliphoridae), the typical initial colonizers of carrion, are attracted to the process of decay. Because of the temporal proximity of death and the subsequent colonization of carrion by blow flies, development data can be used to determine a min PMI by implementing the knowledge about temperature dependency of insect development [5,6,7].

In entomological casework, quantitative evaluation of developmental (e.g., the reaching of the post-feeding stage) and morphological landmarks (like the change in length of the larvae) are combined. Applying published reference data for a variety of insect taxa, mainly blow flies, enables the expert to estimate the age of the immature stages, hence the min PMI of their food source, the human cadaver [2,4,8,9,10,11].

However, morphological methods for estimating the age of juvenile insects can be only of limited use, especially if the oldest specimen found at the crime scene is already a pupa. Here, the standard procedure for min PMI estimations is to rear such specimens under controlled laboratory conditions until the eclosion of the adult fly [8]. Age of a pupa may be reconstructed then by subtracting the time from sampling to eclosion from the published data for total development time. This approach is time-consuming and relies on rearing opportunities, or just can’t be applied when only preserved (this is, killed) samples are available, or relevant specimens die during rearing. Establishing a quick and reliable method for the age estimation of blow fly pupae is therefore still a challenge of current research and particularly important because this stage occupies about 50% of the immature development. By having such a method for determining pupal age, a possibility of staging the fly metamorphosis would have great potential for extending and improving the possibilities of the age determination and thus the min PMI estimation in forensic entomology.

The morphological changes during metamorphosis take place inside an opaque puparium and are not visible from the outside [12,13,14,15,16]. Morphological landmarks can only be examined through a laborious and time-consuming preparation of the insect. The evaluation of the described morphological changes of the insect inside the puparium is strongly dependent on the examiner and hard to quantify even though some attempts have been made by Zajac and Amendt [17], Richards et al. [18], Brown et al. [19] and Martín-Vega et al. [20]. During the last decade, gene expression analysis became increasingly prominent in forensic entomology. First attempts using differentially-expressed genes examining developmental gene expression profiles have been made [21,22,23,24,25,26]. It was assumed that patterns of differentially-expressed genes could help to draw conclusions to the age and therefore the min PMI. Boehme et al. [21,22] were the first to present genetic markers in combination with a statistical tool, i.e., blow fly age calculator (BLOWFLAC), that enables an age prediction of *Calliphora vicina* Robineau–Desvoidy 1830 (Diptera: Calliphoridae) specimens during the metamorphosis based on gene expression analysis using inverse prediction from a standard least-squares regression. In three blind studies, the estimated minimum age approximated the true age by at least 1.0 ± 1.6 days (median ± standard deviation) and the estimated maximum age approximated the true age by up to 1.6 ± 1.7 days. In particular, the precision of age predictions was heavily related to the actual age of the specimens. Most notably, the precision of age predictions is hampered due to a weak accuracy during the first half of the pupal development. The mentioned studies show one thing in common: a high variance and relatively low changes in gene expression during the course of development. In particular, the low expression changes during development hamper a precise molecular age prediction because there are no sharp boundaries between different ages.

Nevertheless, these studies have demonstrated that gene expression analyses can provide quantitative measurements and highly informative conclusions regarding the age of the analysed specimens. Tarone et al. [27], referring to the work of Boehme et al. [22], stated that *improvements in pupal age prediction are possible, if the right loci are chosen for analyses*. With the current study, this thought has been addressed and the identification of the *right* loci was aimed. We generated de novo transcriptomes of 15 different development landmarks of a non-model organism, the blow fly *C. vicina*, during the metamorphosis using the massive parallel sequencing (MPS) technique massive analysis of complementary DNA ends (MACE) [28].

With this approach, marker selection for non-model organisms has been revolutionized [29,30,31]. Zajac et al. [28] demonstrated that a total of 111 genes of interest (GOI), out of more than 53,000 transcripts could be detected. GOI are defined as genes whose transcripts exhibit a significantly higher number of copies at a certain day during metamorphosis. This day- or age specific increase must be significantly higher than the interindividual variability in expression. In addition, 4–10 GOI per analysed transcriptome of each defined pupal age have been selected. These GOI are representing potential loci for gene expression markers for 15 distinct landmarks during metamorphosis. The 15 landmarks resulted from the rearing conditions at 17 °C, since metamorphosis lasts 15 development days and samples were taken every 24 h. Each of those 15 development days represents a developmental landmark.

The current study presents selected markers and the evaluation of a quantitative polymerase chain reaction (qPCR) system. These markers indicate certain landmarks of the physiological age in accumulated degree days (ADD) within metamorphosis. For PMI estimation, these physiological ages have to be translated in absolute age by calculational implementation of the thermal conditions at the scene. The established markers have been tested at pupae raised not only at 17 °C but also at 20 °C and 25 °C to investigate a potential temperature dependent variation of expression.

## 2. Materials and Methods

### 2.1. Specimens

Specimens were obtained from the established stocks of adult *C. vicina* at the Institute of Legal Medicine in Frankfurt am Main, Germany. All colonies are being refreshed in a continuous mode with individuals collected at corpses or in outdoor traps, which occurs every 4–8 weeks. Species identification, rearing and sampling of the specimens was conducted as previously described in Zajac et al. [28]. Oviposition was allowed for 4 h. Sampled specimens were reared in an incubator at a constant temperature of 17 °C, 20 °C or 25 °C, respectively. Sampling was conducted every 24 h after pupariation, and five specimens were arbitrarily preserved per sampling.

It should be noted that, despite the current discussion and the fact that the terminology is actually more complex, the term *pupa* always refers to the entire intra-puparial period in the current article [14].

### 2.2. Identification of Genetic Markers for Age Determination

Markers of age-related genes were identified from MACE data as previously described in Zajac et al. [28]. GOI were defined as transcripts showing a massive, 100 to 1000 fold higher number of normalized transcript counts for a certain day compared to the expression in white pupa as well as to the other days during pupal development. For each analysed age, two markers showing the highest fold change compared to all remaining ages and the sharpest demarcation to the expression from samples taken 24 h prior and 24 h after the actual sample were selected for the current validation study. Additionally, two transcripts showing a constant number of normalized counts at all analysed ages have been identified as reference genes [28].

Markers were initially identified and designed on specimens reared at 17 °C. At this temperature, metamorphosis lasts 15 days, leading to 15 different development landmarks, so far called T1–T15. Since the development of flies is temperature dependent, it is usually converted to a physiological age, namely ADD/accumulated degree hours (ADH). These are calculated from the product of the average environmental temperature and the required development time in days or hours, corrected by the lower development threshold, which is the minimal temperature at which this species may successfully develop. To see whether the molecular age markers correspond with the physiological age at different temperatures, the markers T1–T15 (17 °C) have been adopted to this and renamed in Marker A–O. Note that the amount of required ADD is also dependent on the rearing temperature (Table 1 and Table 2).

Marker A was taken as calibrator for calculation of the fold change (FC) value of Markers B to O. Pupae representing landmark A, the white prepupal stage, still resemble the irreversibly contracted third-instar larvae and are therefore easily distinguishable from all other phases of pupal metamorphosis. Consequently, only 14 markers (B–O) were used for age determination.

### 2.3. Isolation of Total RNA

Isolation of total RNA from entire specimens and subsequent digestion of eventually co-extracted genomic DNA (gDNA) was conducted using TRI-Reagent^®^ as described in Zajac et al. [28]. RNA concentration and purity have been measured in a NanoDrop ND1000 (Thermo Fisher Scientific, Wilmington, DE, USA). Samples were diluted to 10 ng/µL and aliquoted for all qPCR analyses. Each sample was processed independently.

So far, only a few of the explored transcripts have been annotated to known genes, so biological information is lacking. Any co-extracted gDNA can therefore not be avoided by an intron-spanning primer design and has to be removed by DNA digestion prior reverse transcription (RT) qPCR using TURBO DNA-free™ (Ambion, Carlsbad, CA, USA) following the manufacturer’s protocol.

### 2.4. Primer Design

Based on the mentioned MACE data primers for each of the 15 landmarks including the white prepupa were designed using Primer3Plus software [33]. Primer sequences, length of the generated amplicons, annotations to *Drosophila* and general insect data sets in GenBank (http://www.ncbi.nlm.nih.gov/genbank/) (basic local alignment search tool (BLASTX)) and UNIPROT (http://www.uniprot.org/) database if available and the contig number according to the corresponding sequence read archive (SRA) code (SRX977601–SRX977615), respectively, are shown in Table 3. Note that not all of these markers are annotated to known gene products, which is insubstantial for their eligibility as age markers.

### 2.5. One-Step Quantitative Polymerase Chain Reaction

OneStep RT-qPCR was performed using the Promega GoTaq 1-Step RT-qPCR System (Promega Corporation, Madison, WI, USA) for a part of the pupae reared at 17 °C or the Invitrogen EXPRESS One-Step SYBR GreenER™ Kit (Thermo Fisher Scientific) following the manufacturer’s instructions. Comparability of both kits was randomly tested and approved on five samples. RT-qPCR was performed in triplicates for every sample in a 10 µL reaction volume, containing 40 ng total RNA, 200 nM of each primer, 0.2 mL of RT Mix, 30 mM reference Dye (5-Carboxy-Rhodamin-X (ROX) or 6-carboxy-X-rhodamine (CRX)). A no-template control was run in each experiment. RT-qPCR was performed in a StepOnePlus™ Real-Time PCR System (Applied Biosystems^®^, Foster City, CA, USA), using default SYBR^®^ Green Fast thermal cycling conditions with an additional reverse transcription step (50 °C for 5 min) placed in front.

To assess whether the designed qPCR assays produce specific products, melt curve analysis and fragment evaluation on agarose gels were conducted. In some cases, melt curve analysis showed a secondary unspecific peak. These peaks were only detectable at time points where the expression of the analysed amplicons was expected to be very low. When gene expression was high, only specific melt curve peaks were visible. Electrophoresis on agarose gels of all qPCR assays confirmed single bands at the expected product size 

### 2.6. Gene Expression and Data Analysis

Expression of the described markers was normalized to reference genes R1 and R2 (Table 3). DataAssist™ Software v3.01 from 2012 (Applied Biosystems) was used to calculate the relative quantification (RQ = FC) of gene expression, using the comparative Cq (ΔΔCq) method [34]. Specimens sampled on day one (landmark A) were easily distinguishable from the other sampled specimen as they are still in the so-called white prepupal stage. Therefore, in the current study, gene expression in these specimens was used as a calibrator at each temperature regime, respectively, to calculate relative gene expression (FC) of the older pupae.

Data visualization and statistical analysis were performed using GraphPad Prism (version 7.00 for Windows, GraphPad Software, La Jolla, CA, USA, www.graphpad.com). For visualization, data are presented in Tukey plots.

Fold change values were subjected to a D’Agostino–Pearson normality test and subsequently analysed by one-way analysis of variance (ANOVA) followed by an uncorrected Fisher’s least significant difference (LSD) test or Kruskal–Wallis test followed by an uncorrected Dunn’s test, respectively (*p*-value < 0.05), to explore the influence of age on gene expression of the examined markers, which was treated as a factor. Comprehensive full statistical analysis is available on request.

## 3. Results and Discussion

By virtue of the constant interest in establishing more and more precise methods to determine the age of juvenile blow flies, i.e., blow fly pupae, using gene expression was focused in recent years [21,22,23,24,25,26,35]. However, these approaches were mainly hampered by high interspecific variability compared with age specific differences. This now has been overcome by the newly identified marker transcripts. These markers are suitable for detecting a certain age by showing mostly a single specific up-regulation for a short period during metamorphosis regardless of the rearing temperature.

**Figure 1:**
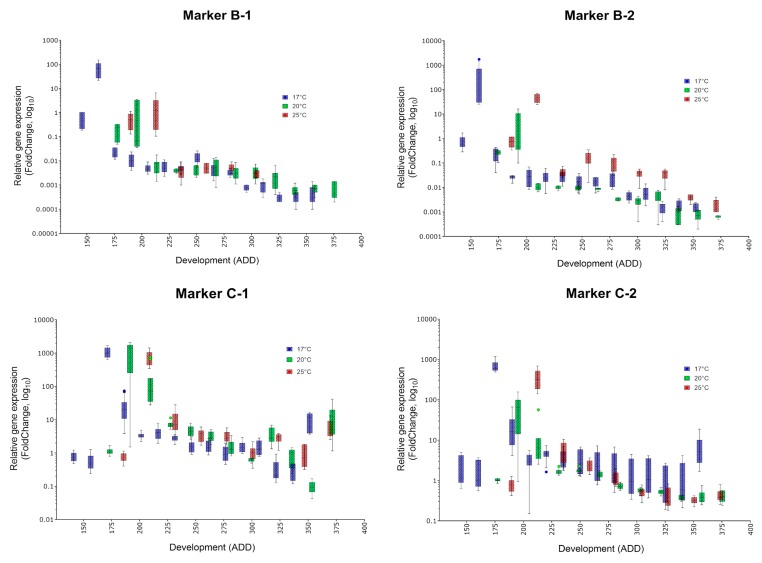

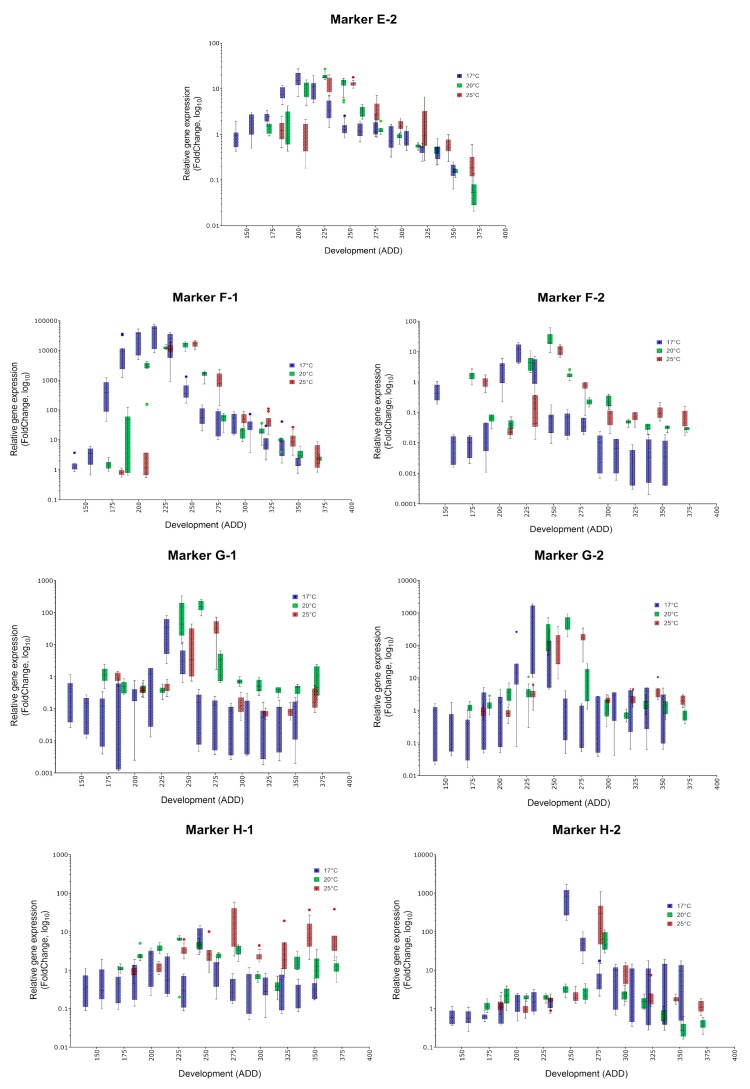

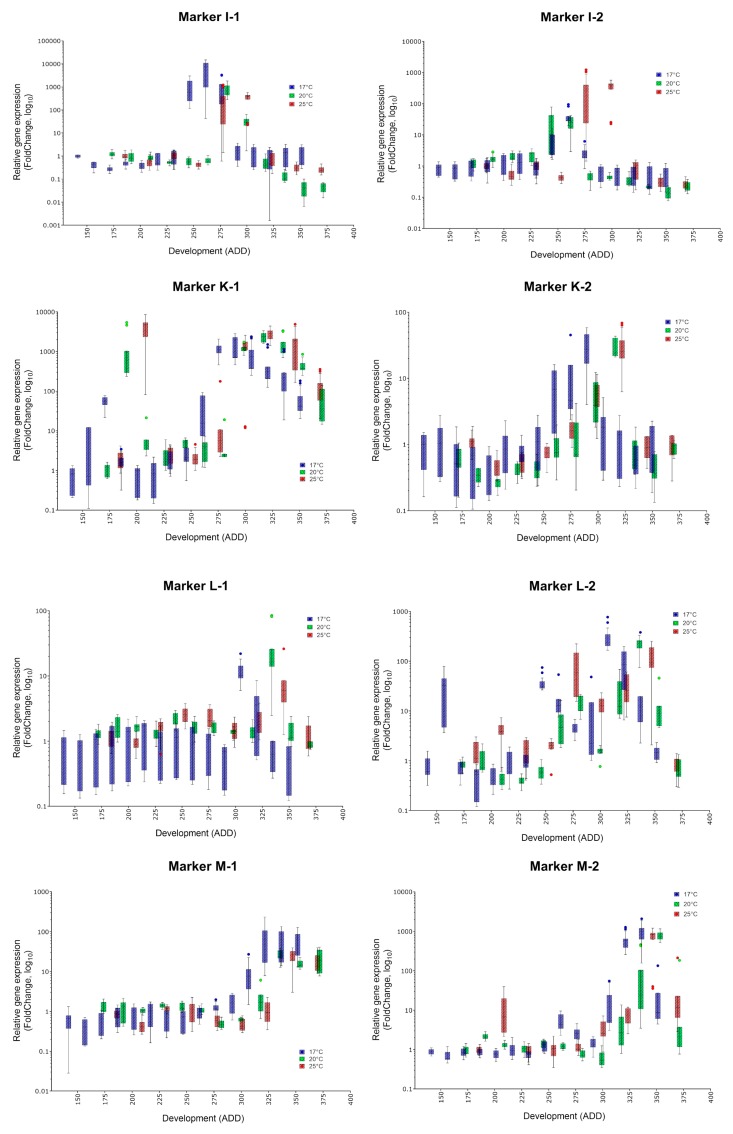

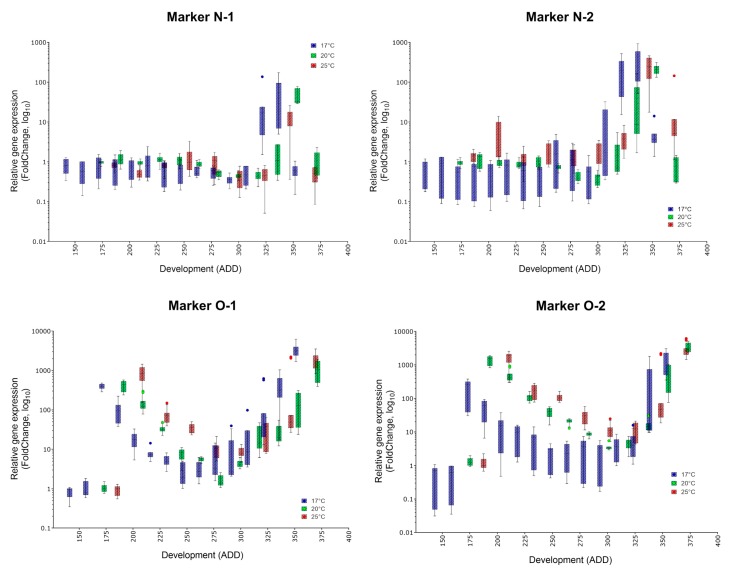
Tukey plots visualizing the expression level of differentially genes showing a demarcated area of higher gene expression during metamorphosis. Fold change (FC) values are plotted against pupal age (ADD, temperature threshold for development 2 °C) Blue boxes represent data from the 17 °C study, green boxes represent 20 °C data and red boxed represent 25 °C data. First sampling at each temperature represents the white prepupal stage, at which a FC of one would occur when Marker A is applied (not depicted).

Gene expression patterns were investigated by qRT-PCR, i.e., two markers for each of 14 development landmarks (compared to Marker A) within metamorphosis to raise the chance of detecting suitable markers for every physiological age in ADD. Figure 1 represents a graphical description of all markers deployed at each temperature, which demonstrates the high age specific expression changes compared to the interindividual variation. Note that there is a shift in the maximal expression of each marker dependent on the rearing temperature applied, due to the fact that the required ADD is temperature dependent (Table 1).

In contrast to the markers published so far (e.g., [21,24,25]), the new transcripts show that their expression during the investigated pupal ages is characterized by a quick change of expression during development, which is important in terms of high predictive power. However, interreplicate variance, especially at low temperatures (17 °C), is also quite high in the expression within our study, e.g., Figure 1 marker G-1, G-2. However, the markers show a massive change in gene expression within the developmental landmarks, which counterbalance the observed variance. Most notably, and in contrast to other studies, almost all of our markers detected an extreme change in gene expression [21,22,24,26]. Fold change values between approximately 10 and 40,000 compared to their expression immediately after pupariation (so called white prepupa) are achieved (Figure 1).

To determine whether age has significant influence on the expression level of the established markers, either Kruskal–Wallis test or ANOVA were used. Subsequently, multiple comparison tests were conducted to explore significant differences between the tested groups (Table S1). A group is defined as all samples of a certain time or the same age, respectively.

Table 4 depicts the significance of the variance of each marker expression changes compared to the expression status in white prepupae. Most markers exhibit no overlap between the corresponding and the other states and are beneficial in terms of age determination.

**Figure 2:**
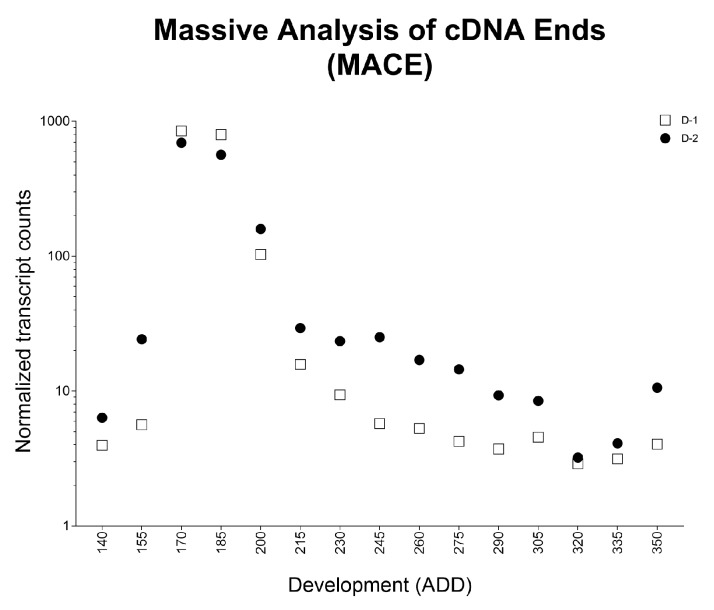
Normalized transcript counts of contigs identified for the markers D-1 and D-2 in the previous massive analysis of complementary DNA ends (MACE) study (17 °C) [28] plotted against pupal age (ADD).

**Figure 3:**
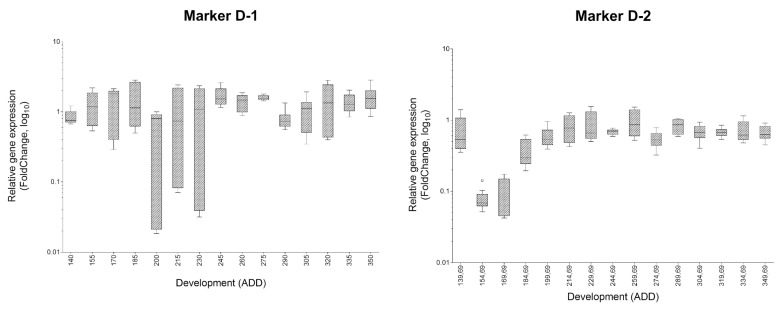
Tukey plots visualising the expression level of markers D-1 and D-2 showing no demarcated area of higher gene expression during metamorphosis under a temperature regime of 17 °C. FC values are plotted against pupal age (metamorphosis) in ADD. First sampling at 140 ADD is defined to be the first day of metamorphosis (white prepupa) and data were used as calibrator for FC calculation.

However, some markers show overlaps. While the two markers for landmark B and C (approximately first quarter of development) and for landmark F to O (approximately second and third trimester of development) exhibit a highly significant disparity in each applied temperature, qPCR data of D-1 and D-2 at 17 °C are not congruent with the corresponding data explored by MACE (Figure 2). Here, between 566 and 849 counts of these markers at a developmental progress of 170–207 ADD have been observed compared to an average count of 13 or 25, respectively, at the remaining development landmarks [28]. In the qPCR analysis, however, a totally different pattern of gene expression during development is observed (Figure 3). Therefore, they have been excluded from analysis also at 20 and 25 °C.

Another inconclusiveness has been observed concerning marker E-1. Here, we did not achieve reproducible results in qPCR, therefore the marker is excluded from further analysis.

Markers that show an ambiguous behaviour are those that shall be indicated within the first trimester of pupal development. This leads to the assumption that a biological impact takes place. A hypothesis for this observation is that gene activity is low during this period as the number of genes exclusively up-regulated during this period decreased compared to other development phases [28,36,37]. Moreover, genes up-regulated during this period meet the requirements for GOI at the lower limits as they have been described in Zajac et al. [28]—markers D-1 and D-2 do belong to this period. Interestingly, morphological approaches highlighted that only some minor changes in the shape of the pharate adult and the internal morphology of *C. vicina* specimens, such as the volume of the alimentary canal and the histogenesis of adult flight muscles, were observed during the mentioned period [17,20].

Regardless of the statistical data, as discussed so far, both markers of each landmark are not always equally well suited. Table 5 provides a summary of the applicability of the investigated markers. It can be seen that some of the presented markers show a second expression peak at another *wrong* development phase (H-1, J-1, K-1, L-2, O-1 and O-2). Multiple comparison analysis of these markers confirms either still significant differences between both peaks or the optical impression of lower significance (Table S1). Average FC values for the actual and secondary peaks, if they occur, are shown in Table 5. In general, although secondary peaks having similar amplitudes may interfere with age determination, these markers should nevertheless be used because they can provide additional information in combination with the other markers.

Marker H1 has a secondary peak in breeding at 20 and 25 °C, not at those developed at 17 °C. This indicates a potential temperature dependent expression of this marker at this age, which makes this marker inappropriate for age estimation in a forensic context. Furthermore, besides the described temperature dependency, the change in gene expression of marker H-1 in the course of metamorphosis is very low compared to marker H-2 and markers for other ages. The application of marker H-1 could produce ambiguous results and therefore it is not recommended for the use in age predictions. Here, only marker H-2 provides sufficient informative data for age determination.

Irrespective of the rearing temperature, nearly all of the presented markers show a significant increase of gene expression at a certain physiological time in ADD during development. Temperature dependent deviation observed in Marker H1, a so far uncharacterized transcript, illustrates that temperature sensitivity can be a problem for interpretation of gene expression data. Therefore, temperature sensitivity has to be taken into account also for detection of new markers as well as validation studies, preferably already on the level of MACE.

The temperature dependency of the required heat amount (Table 1, [38,39]) is indicated in Figure 1 in the way that each marker shows its individual expression maximum at slightly other ADD values. This shift emphasizes the importance of implementing the temperature during development in the analysis of the markers because markers highly expressed in the pupa indicate different ADD values due to the temperature regime. However, the mandatory reconstruction of the temperature regime is a common procedure in forensic entomology also when morphological characteristics are used.

For the presented markers, further validation has to be undertaken for temperatures other than 17–25 °C prior usage outside this corridor. This is important for the usability in casework, especially because the performance of these markers in the designed qPCR assays is not quite as sharp as the corresponding data generated by MACE. Due to a faster pupal development at higher temperatures, two markers consecutive in indicating development can be expressed simultaneously in certain scenarios. Because temperature reconstruction at the scene is mandatory in entomological casework due to the temperature dependent development of insects, this can be implemented in the overall analysis. Equivalent data handling must be performed at temperatures below 17 °C where a certain segment of metamorphosis may last more than one day. Furthermore, mathematical models have to be developed to estimate the variance of age determination, which include the temperature dependent ADD values needed for development.

In case work scenarios, we do not deal with the described homogeneous temperature conditions. There we have fluctuating temperature profiles, but the general congruency during the course of metamorphosis is promising to withstand also a validation applying fluctuating temperatures (currently in progress).

## 4. Conclusions

Beneath the observation that the expression of eligible markers do not only exhibit highly significant changes compared to the status in white pre-pupae, the fact that an overlap of gene expression status of these markers compared with their expression at non corresponding landmarks is very limited makes the system utilizable in a forensic context where often the question arises if a certain scenario (i.e., age of an immature insect) can be excluded—in contrast to its improbability.

For application of molecular age markers in forensic entomology, it has to be stated that, for most of the defined developmental landmarks of *C. vicina* (except landmarks D and E), qPCR markers could be established that depict the corresponding physiological age in ADD, within a temperature range between 17 and 25 °C. Until now, this *digital* age detection using the presented qPCR assays has not yet been applied for entomological casework. 

With regard to an application, the following workflow should be adhered to:

- Ideally, RNA should be extracted as soon as possible after asservation. If the samples cannot be processed immediately, they should be preserved in 70–90% Ethanol or Trizol [40] to avoid significant RNA damage.
- The indicator of a certain age is a massive increase of the corresponding marker compared to stage A, the white prepupa (and most other phases of metamorphosis). Thus, all presented eligible markers should be typed in order to gain a detailed overall impression.
- Since development and the indicated ADD value is temperature dependent, the occurrence of a highly expressed marker must be translated into absolute age. For *C. vicina* and many other forensically-relevant blow fly taxa, reference data on the duration of development and the required ADD values at different constant temperatures since oviposition exist [8,38,39].

Overall, it has become obvious that these new markers established based on transcriptome data will contribute to more precise min PMI estimation compared to so far published markers for molecular age determination.

## Figures and Tables

**Table 1:** Minimum (min) and maximum (max) development periods in accumulated degree days (ADD) and in (days) of *Calliphora vicina* during this study. For ADD calculation, a constant lower development threshold of 2 °C for all three rearing temperatures was assumed [32].

	Oviposition to Pupariation (White Prepupae)		Pupariation to Adult Emergence		Oviposition to Adult Emergence	
Temp.	min	max	min	max	min	max
**17 °C**	132.7 (8.8)	150.9 (10.1)	207.2 (13.8)	242.6 (16.2)	358.1 (23.9)	375.3 (25.0)
**20 °C**	157.1 (8.7)	180.4 (10.0)	181.1 (10.1)	238.9 (13.3)	361.5 (20.1)	396.0 (22.0)
**25 °C**	178.3 (7.8)	189.8 (8.3)	194.0 (8.4)	216.1 (9.4)	383.8 (16.7)	394.4 (17.1)

**Table 2:** Sampling days assigned to the progress of metamorphosis in ADD and the corresponding gene expression marker for breeding at 17, 20 and 25 °C. Each of those 15 development days represents a developmental landmark.

17 °C			20 °C			25 °C		
Sampling Day	ADD	Marker	Sampling Day	ADD	Marker	Sampling Day	ADD	Marker
1	140	A	1	172	A/B	1	184	A/B
2	155	B	2	190	B/C			
3	170	C				2	207	B/C/D
4	185	D	3	208	C/D			
5	200	E	4	226	D/E	3	230	D/E
6	215	F	5	244	E/F/G	4	253	E/F/G
7	230	G	6	262	G/H			
8	245	H	7	280	H/I	5	276	G/H
9	260	I				6	299	I/J
10	275	J	8	298	I/J			
11	290	K	9	316	J/K/L	7	322	J/K/L
12	305	L	10	334	L/M	8	345	L/M
13	320	M	11	352	M/N			
14	335	N				9	368	N/O
15	350	O	12	370	O			

**Table 3:** Markers and primer sequences used for analysis of *C. vicina* pupae in this study. Contig aliases and annotations are given for reference. Annotated via basic local alignment search tool (BLAST) assembly mapping against *Drosophila* and general insect data sets available in GenBank (BLASTX) and UNIPROT database [28]. Massive analysis of complementary DNA ends (MACE) count numbers of the used transcripts are indicated against landmark A (white prepupa) and in comparison to the mean count numbers at all other developmental landmarks.

Marker	Forward Primer	Reverse Primer	Product Length (bp)	Normalized Transcript Counts (Landmark A)	Mean Normalized Transcript Counts of Other Landmarks	Contig	Annotation
B-1	ACATCTCCGCTCGCATTCTCC	CGTGTAACCAAGCTCCGCATT	164	3837.134 (68.34)	10.869	Contig5316	tr|B4LCU1|B4LCU1_DROVI GJ12906 OS = *Drosophila virilis* GN = Dvir\GJ12906 PE = 4 SV = 1
B-2	TCTTGGGTGCAGGACGACATT	GGGTAGACTCTCGTCGTTGTG	72	4168.19 (77.346)	13.423	Contig5717	tr|Q9W4B7|Q9W4B7_DROME CG4151 OS = *Drosophila melanogaster* GN = CG4151 PE = 2 SV = 1
C-1	CCAGCTGCCGTTACTCCTTATC	CCGTAGACTTCATCGGGTTGTT	104	457.333 (0.795)	2.692	Contig3033	tr|T1PL89|T1PL89_MUSDO Uncharacterized protein OS = *Musca domestica* PE = 2 SV = 1
C-2	GATGAAGTCTACGGCCCACC	CTGCGAGCAGATGAAGTACGG	191	353.407 (0.662)	2.450	Contig3033	tr|T1PL89|T1PL89_MUSDO Uncharacterized protein OS = *Musca domestica* PE = 2 SV = 1
D-1	ATGTCGAGGTCCACCTGTAGT	AACAAACTGGGCGAAGGGAA	114	798.587 (3.973)	72.970	Contig3088	sp|O76217|PE1_ANOGA Peritrophin-1 OS = *Anopheles gambiae* GN = Aper1 PE = 2 SV = 2
D-2	CCAGCCGAGTTCTAATGCCC	GTTTCGCCTTTGGTCGTTCC	103	566.234 (6.357)	73.474	comp162812_c0_seq1	uncharacterized RNA
E-1	ACCGGACGTCACTGATTCCTT	AGCGTTATGCTGGTCTGGTC	171	46.499 (14.039)	3.796	Contig5877	sp|Q7JQ07|MOS1T_DROMA Mariner Mos1 transposase OS = *Drosophila mauritiana* GN = mariner\T PE = 1 SV = 1
E-2	CTCATTGCATTGGCAGAGAGACTG	AAAGCACCCACCGATGACAG	128	261.985 (13.377)	41.105	Contig2811	uncharacterized RNA
F-1	GGCCTTAAGCTCTAATTGTCCCTC	CTTGATATTGCCGGAGCCCA	163	14612.462 (53.109)	1564.769	Contig2939	uncharacterized RNA
F-2	AGGACAGTTGATGTCCGGTTTC	GCTAGACATGGTGTGAATTCGGG	129	1789.857 (113.9)	93.082	Contig5446	uncharacterized RNA
G-1	ACGTTGACAAGTGTCTGGCTC	CTGGCTATGACGCTCTCGCA	179	276.185 (0.795)	6.565	comp11460_c0_seq1	tr|S6B1X7|S6B1X7_9MUSC Odorant-binding protein OS = *Delia antiqua* GN = DantOBP16 PE = 2 SV = 1
G-2	AGCCCAATACGAAGGAGCCA	CAGTCAGCCATCGTTCCATTCTTG	159	931.119 (2.914)	32.261	Contig1423	tr|B4M6C9|B4M6C9_DROVI GJ10414 OS = *Drosophila virilis* GN = Dvir\GJ10414 PE = 4 SV = 1
H-1	GGGCTATTCTACACATCATACGGG	ACCAAGACCGTGAGCCTGTT	115	150.791 (0.265)	0.348	comp16284_c0_seq1	uncharacterized RNA
H-2	CCGCCCTGATAGCAATTATAGTCC	TCTGAGACTTAGTGCGCTGTCC	176	383.904 (1.06)	3.586	comp19725_c0_seq1	uncharacterized RNA
I-1	AAGAAACGCTCTGGACGCAA	CGAGCCAAGAATGGAGGTGG	107	1698.144 (3.708)	45.997	comp13408_c1_seq1	uncharacterized RNA
I-2	GCGGTGCCCAACTACCAAATAA	TACTGACACCACTTAGACCCGA	192	543.64 (0.662)	14.865	comp10716_c0_seq1	tr|B4IZK1|B4IZK1_DROGR GH17053 OS = *Drosophila grimshawi* GN = Dgri\GH17053 PE = 4 SV = 1
J-1	CCAGGAGGGCAAATGTAGACCA	TCCATACCCACTGCCGTTTC	177	1542.921 (5.165)	154.044	Contig1789	tr|B4MN60|B4MN60_DROWI GK17641 OS = *Drosophila willistoni* GN = Dwil\GK17641 PE = 4 SV = 1
J-2	TCACCATCTCTGGTCTCCCAA	GCTCATGAGGATTATGAGGGTGG	134	1164.349 (3.311)	112.076	Contig4584	sp|P45583|CU19_LOCMI Cuticle protein 19 OS = *Locusta migratoria* PE = 1 SV = 1
K-1	GGAGAAGACAGGACAGACTTGG	CAAGCCGCCAAACAATACGG	150	2137.504 (10.066)	267.367	Contig5672	gi|296335865|gb|EZ599050.1| TSA: *Sarcophaga crassipalpis* HAHN.FLY.1245.C4 mRNA sequence///tr|B4MMM7|B4MMM7_DROWI
K-2	CGAGTGGGTGGCAACAAGAA	CCATACGCGAAGTTCCGACA	166	239.901 (4.371)	12.681	Contig3509	sp|P05804|BGLR_ECOLI Beta-glucuronidase OS = *Escherichia coli* (strain K12) GN = uidA PE = 1 SV = 2
L-1	TGTTGACACTGGCGAAGTGGA	CTACGCTCGCCTTCTACATCATC	144	545.983 (2.119)	51.450	Contig4745	uncharacterized RNA
L-2	CCGCTAGGAGCAGAAGGTAGT	CCTCCATCAGGTGTAGGAAGTGA	197	480.257 (3.576)	59.589	Contig421	tr|B3MYH7|B3MYH7_DROAN GF22144 OS = *Drosophila ananassae* GN = Dana\GF22144 PE = 4 SV = 1
M-1	ATTCACAAACCGGCAAGGGT	GCCAGCAGTGTAGGAGCAAA	173	289.837 (0.795)	33.279	comp21483_c0_seq1	sp|Q8MUG0|GPL_GLOFF Lectizyme OS = *Glossina fuscipes fuscipes* GN = Gpl PE = 2 SV = 1
M-2	GCAACAATGGGAGCAGCAAC	TTGACCGGTGACAGCAAGAG	179	708.812 (3.046)	106.042	Contig4395	sp|P81225|CU21_LOCMI Cuticle protein 21 OS = *Locusta migratoria* GN = ACP21 PE = 1 SV = 1
N-1	GAGCAGCACAAGCCAATCTCT	ACATAATAAGGACGCCACGCTC	157	128.003 (1.192)	5.173	comp5434_c0_seq1	tr|B3MIB5|B3MIB5_DROAN GF12734 OS = *Drosophila ananassae* GN = Dana\GF12734 PE = 4 SV = 1
N-2	AAACGAGCGGGTACAGCCA	GGGTTCCTACTCCGTTGTAGATG	174	1203.364 (3.046)	67.999	Contig4395	sp|P81225|CU21_LOCMI Cuticle protein 21 OS = *Locusta migratoria* GN = ACP21 PE = 1 SV = 1
O-1	GCTTTGTGCTTGTTGGCTGTTG	AGGCTGTGGTGTAGGGTGAAG	82	512.401 (1.722)	3.453	Contig3447	uncharacterized RNA
O-2	TGTAGGCAGCAGTGTAGGGA	CGCTATTGTTGCCTTGGCTG	98	1005.226 (2.914)	34.484	comp21535_c2_seq20	gi|296343591|gb|EZ606776.1| TSA: *Sarcophaga crassipalpis* HAHN.FLY.7765.C7 mRNA sequence
R1*	AAGGTGGTGGCGGTTGATTT	TCGACCACAGGTGGAGAAGA	175	Mean norm transcript counts (SD) 127.799 (19.657)		Contig3750	tr|T1P7X0|T1P7X0_MUSDO Uncharacterized protein OS = *Musca domestica* PE = 4 SV = 1
R2*	ACAATGGCCGCCTTCCTTGA	AGGATCCTCCAGTCCGCAAG	83	Mean norm transcript counts (SD) 137.088 (15.225)		Contig4663	sp|P53575|ETFB_BRAJA Electron transfer flavoprotein subunit beta OS = *Bradyrhizobium japonicum* (strain USDA 110) GN = etfB PE = 3 SV = 2

* Reference genes.

**Table 4:** One-way analysis of variance (ANOVA) and the Kruskal–Wallis test for changes in gene expression during metamorphosis. Markers D1 and D2 showed negligible change of gene expression at 17 °C and were not analysed at 20 °C and 25 °C. Marker E1 qPCR assay failed to detect gene expression.

	17 °C				20 °C				25 °C			
Marker	Test	χ2 or F Value	*p* Value	R^2^	Test	χ2 or F Value	*p* Value	R^2^	Test	χ2 or F Value	*p* Value	R^2^
B-1	ANOVA	343.3	<0.0001	0.9581	Kruskal–Wallis	122.4	<0.0001	-	Kruskal–Wallis	73.83	<0.0001	-
B-2	Kruskal–Wallis	194.6	<0.0001	-	Kruskal–Wallis	139.2	<0.0001	-	ANOVA	86.11	<0.0001	0.8548
C-1	Kruskal–Wallis	177.2	<0.0001	-	Kruskal–Wallis	151.6	<0.0001	-	ANOVA	194	<0.0001	0.9249
C-2	Kruskal–Wallis	119.6	<0.0001	-	ANOVA	58.9	<0.0001	0.8029	Kruskal–Wallis	115.8	<0.0001	-
D-1	ANOVA	3.061	0.0005	0.2681	-	-	-	-	-	-	-	-
D-2	ANOVA	38.82	<0.0001	0.82229	-	-	-	-	-	-	-	-
E-1	-	-	-	-	-	-	-	-	-	-	-	-
E-2	Kruskal–Wallis	167.2	<0.0001	-	Kruskal–Wallis	167.8	<0.0001	-	ANOVA	66.75	<0.0001	0.8091
F-1	Kruskal–Wallis	205.6	<0.0001	-	Kruskal–Wallis	158.2	<0.0001	-	Kruskal–Wallis	119.3	<0.0001	-
F-2	Kruskal–Wallis	177	<0.0001	-	ANOVA	469.3	<0.0001	0.969	ANOVA	81.16	<0.0001	0.8375
G-1	Kruskal–Wallis	102.4	<0.0001	-	Kruskal–Wallis	119.3	<0.0001	-	Kruskal–Wallis	117.8	<0.0001	-
G-2	Kruskal–Wallis	104.4	<0.0001	-	Kruskal–Wallis	132.1	<0.0001	-	ANOVA	132.6	<0.0001	0.8938
H-1	Kruskal–Wallis	70.01	<0.0001	-	Kruskal–Wallis	127.4	<0.0001	-	Kruskal–Wallis	84.19	<0.0001	-
H-2	Kruskal–Wallis	115.9	<0.0001	-	Kruskal–Wallis	144.4	<0.0001	-	ANOVA	88.37	<0.0001	0.8487
I-1	Kruskal–Wallis	146.5	<0.0001	-	ANOVA	299.7	<0.0001	0.9532	ANOVA	111.5	<0.0001	0.8788
I-2	Kruskal–Wallis	118.7	<0.0001	-	Kruskal–Wallis	142.6	<0.0001	-	Kruskal–Wallis	115.2	<0.0001	-
J-1	Kruskal–Wallis	209.6	<0.0001	-	Kruskal–Wallis	163.5	<0.0001	-	ANOVA	139.2	<0.0001	0.8983
J-2	Kruskal–Wallis	124.5	<0.0001	-	Kruskal–Wallis	131.2	<0.0001	-	ANOVA	271.2	<0.0001	0.9476
K-1	Kruskal–Wallis	182.3	<0.0001	-	ANOVA	392.8	<0.0001	0.9639	ANOVA	373	<0.0001	0.9623
K-2	Kruskal–Wallis	108.5	<0.0001	-	Kruskal–Wallis	106.1	<0.0001	-	Kruskal–Wallis	99.4	<0.0001	-
L-1	Kruskal–Wallis	71.46	<0.0001	-	Kruskal–Wallis	81.5	<0.0001	-	ANOVA	22.24	<0.0001	0.5913
L-2	Kruskal–Wallis	187.9	<0.0001	-	Kruskal–Wallis	156.4	<0.0001	-	Kruskal–Wallis	100.7	<0.0001	-
M-1	Kruskal–Wallis	158.6	<0.0001	-	Kruskal–Wallis	137.2	<0.0001	-	ANOVA	118.5	<0.0001	0.8826
M-2	Kruskal–Wallis	148.2	<0.0001	-	ANOVA	145.4	<0.0001	0.9096	ANOVA	115.9	<0.0001	0.8829
N-1	Kruskal–Wallis	87.68	<0.0001	-	Kruskal–Wallis	113.7	<0.0001	-	ANOVA	17.89	<0.0001	0.5318
N-2	Kruskal–Wallis	117.5	<0.0001	-	Kruskal–Wallis	119.3	<0.0001	-	Kruskal–Wallis	73.99	<0.0001	-
O-1	Kruskal–Wallis	191	<0.0001	-	Kruskal–Wallis	159.8	<0.0001	-	Kruskal–Wallis	116.9	<0.0001	-
O-2	Kruskal–Wallis	145.4	<0.0001	-	ANOVA	427.6	<0.0001	0.9679	Kruskal–Wallis	120.7	<0.0001	-

**Table 5:** Summary of the characteristics of the investigated markers regarding their applicability: specific peaks corresponding to the developmental progress they are designed for. Mean FC compared to the white prepupal stage (landmark A). Occurrence of a secondary peak and the mean FC value compared to the white prepupal stage (landmark A). Recommendation for the applicability according to their characteristics: Checkmarks indicate a sharp demarcation of gene expression at the corresponding stage of development. (Checkmarks) indicate a weaker demarcation of gene expression at the corresponding stage of development.

Marker	Specific Peak	Mean FC (17, 20, 25 °C)	Secondary Peak	Mean FC (17, 20, 25 °C)	Applicability
**B-1**	✓	72.5, 1.315, 2.1	no		(✓)
**B-2**	✓	426.1, 5.246, 28.5	no		✓
**C-1**	✓	966.9, 210.6, 763.5	no		✓
**C-2**	✓	684.8, 62.95, 350.8	no		✓
**E-2**	✓	9.2, 19.0, 13.39	no		(✓)
**F-1**	✓	41764, 15390, 16534	no		✓
**F-2**	✓	10.9, 27.5, 12.17	no		(✓)
**G-1**	✓	34.3, 173.1, 34.63	no		(✓)
**G-2**	✓	818, 495.3, 172.4	no		✓
**H-1**	(✓)	7.9, 3.3, 23.5	only in breeds at 20 and 25 °C	9.6, 11.3	-
**H-2**	✓	796.9, 61.9, 347.7	no		✓
**I-1**	✓	6633, 811.6, 348.7	no		✓
**I-2**	✓	44.3, 46.66, 26.05	no		(✓)
**J-1**	✓	2094, 796.6, 2378	yes	42.85, 31.61, 37.39	(✓)
**J-2**	✓	523.1, 93.72, 763.3	no		✓
**K-1**	✓	1617, 2329, 2808	yes	274.5, 1511, 4008	(✓)
**K-2**	✓	28.85, 29.67, 30.58	no		✓
**L-1**	✓	11.86, 28.86, 9.1	no		(✓)
**L-2**	✓	312.7, 216.6, 113	yes	36.7, 14.08, 86.72	(✓)
**M-1**	✓	80.4, 25.68, 22.82	no		(✓)
**M-2**	✓	489.8, 787.4, 711.6	no		✓
**N-1**	✓	53.89, 47.32, 12.94	no		(✓)
**N-2**	✓	344, 211.6, 245.2	no		✓
**O-1**	✓	3384, 1120, 1840	yes	1023, 421.8, 825.2	✓
**O-2**	✓	1288, 3380, 3079	yes	198.8, 1442, 1592	(✓)

## Supplementary Materials

The following are available online at http://www.mdpi.com/2073-4425/9/3/153/s1. Table S1: Multiple comparison (MC) analysis. Raw data S2: Absolute C_T_ values of all markers at all ages.
