# Supplementary material for: Dating Pupae of the Blow Fly Calliphora vicina Robineau–Desvoidy 1830 (Diptera: Calliphoridae) for Post Mortem Interval—Estimation: Validation of Molecular Age Markers
Source: Genes (Basel). 2018 Mar 9;9(3):153. doi: 10.3390/genes9030153 (PMC5867874; doi:10.3390/genes9030153)
Supplement: Supplementary file 1 [file genes-09-00153-s001.zip › S2/Caption S2.docx]

Caption S2.

Raw data - absolute ct values of all markers at all ages of *Calliphora vicina* specimens reared at 17 °C, 20 °C and 25 °C. Exported StepOnePlus run files.

The file names were assigned before the markers were renamed to A-O. The file names reflect the examined marker, the rearing temperature, and the investigated sample days - e.g. PT07-1_CV17G1_01 stands for marker G-1 (PT07-1), *C. vicina* reared at 17 °C (CV17G1), samples from day 1.

For the 17 °C study initially three pupae also used for the previous MACE study were examined. Therefore, there is the addition “_3MACE-Samples” in the file names.

Furthermore, two additional samples were examined to get 5 specimens altogether.

The corresponding files were preceded by "Additional2Samples_" to the actual file name.

Within the files of each temperature study, the samples are referred to as “Sample 01 – XX”.

At 25 °C:

9 sampling days x 5 specimen = 45 measurements.

Sample 01-05 = day 1, Sample 06-10 = day 2, etc.

At 20 °C:

12 sampling days x 5 specimen = 60 measurements.

Sample 01-05 = day 1, Sample 06-10 = day 2, etc.

At 17 °C:

15 sampling days x initial 3 specimen = 45 measurements (MACE pupae)

Sample 01-03 = day 1, Sample 04-06 = day 2, etc.

In addition, 15 x 2 = 30 further measurements
